# Novel Repair Concept for Composite Materials by Repetitive Geometrical Interlock Elements

**DOI:** 10.3390/ma4122219

**Published:** 2011-12-20

**Authors:** Werner Hufenbach, Frank Adam, Thomas Heber, Nico Weckend, Friedrich-Wilhelm Bach, Thomas Hassel, David Zaremba

**Affiliations:** 1 Technische Universität Dresden, Institut für Leichtbau und Kunststofftechnik, Holbeinstr. 3, Dresden 01307, Germany; E-Mails: ilk@ilk.mw.tu-dresden.de (W.H.); f.adam@ilk.mw.tu-dresden.de (F.A.); n.weckend@ilk.mw.tu-dresden.de (N.W.); 2 Leibniz Universität Hannover, Institut für Werkstoffkunde, An der Universität 2, Garbsen 30823, Germany; E-Mails: office@iw.uni-hannover.de (F.-W.B.); hassel@iw.uni-hannover.de (T.H.); zaremba@iw.uni-hannover.de (D.Z.)

**Keywords:** composite repair, fiber-reinforced polymers, positive locking connection

## Abstract

Material adapted repair technologies for fiber-reinforced polymers with thermosetting matrix systems are currently characterized by requiring major efforts for repair preparation and accomplishment in all industrial areas of application. In order to allow for a uniform distribution of material and geometrical parameters over the repair zone, a novel composite interlock repair concept is introduced, which is based on a repair zone with undercuts prepared by water-jet technology. The presented numerical and experimental sensitivity analyses make a contribution to the systematic development of the interlock repair technology with respect to material and geometrical factors of influence. The results show the ability of the novel concept for a reproducible and automatable composite repair.

## 1. Introduction

The increased application of fiber-reinforced polymers in varying industries accounts for adapted repair concepts with respect to the possible kinds of deterioration. Besides repair solutions for surface deteriorations, especially technologies for the reconstitution of the composite properties were developed. All these technologies are mainly based on manually realized mountings in the near field of the deterioration or extensively applied patches, respectively [1,2,3,4]. Besides the mechanical exposing of deteriorated laminate plies, the composite surface can also be ablated thermally by use of laser technology, roughened by means of sandblasting, or embrittled using ultraviolet radiation [5].

In order to reach an entire reconstitution of the part properties, repair techniques with increased compaction pressures are necessary, which can only be achieved by means of autoclave and pressing technologies. Due to the generally high part dimensions and volume, these technologies possess only a limited application for repair. Hence, the repair in practice is currently carried out by vacuum-assisted infiltration of limp soft-patches [6,7,8,9] or by gluing of rigid hard-patches [10,11,12,13,14,15,16,17,18], respectively.

The currently used repair concepts in industry are mainly based on the manual realization of a scarfed intersection from the basic laminate to the repair patch. Due to the minimization of changes in stiffness, the needed scarf angle leads to an extensive repair area, even in cases of small-area damages. Furthermore, an increased risk of deviations in properties of the repair area exists because of the high manual effort. In contrast, overlaps as an alternative repair concept are mainly characterized by a significant raise of the wall thickness and thus are not appropriate for visible surfaces.

## 2. Novel Repair Concept

**Figure 1:**
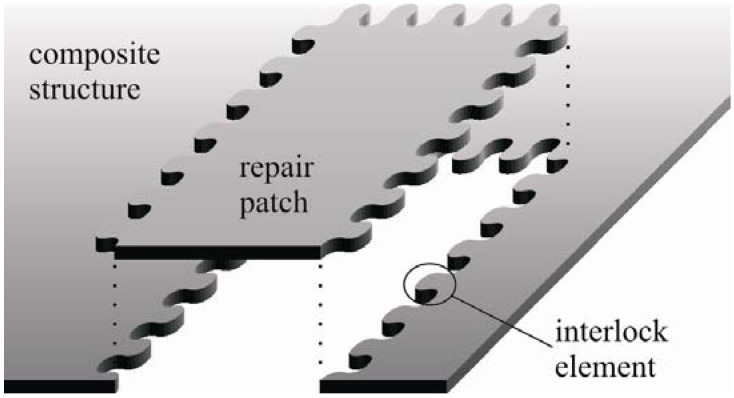
Novel interlock composite repair concept.

Due to their excellent specific properties, fiber-reinforced polymers are increasingly used for applications in highly loaded structures for aviation industry, vehicle construction, shipbuilding, as well as mechanical engineering. Thus, major importance is placed on composite-adapted repair technologies, which allows for a wide preservation and reproducible reconstruction of the mechanical part properties. Therefore, a novel repair technology based on form-closed and adhesive bond interlock elements is investigated [19]. The interlock technology is specifically tailored to a one-sided automated repair zone preparation and subsequent repair of composite structures. Hence, a minimal repair zone even for thick laminates and the preservation of the surface quality becomes possible (Figure 1).

## 3. Preliminary Design and Simulation Studies

**Figure 2:**
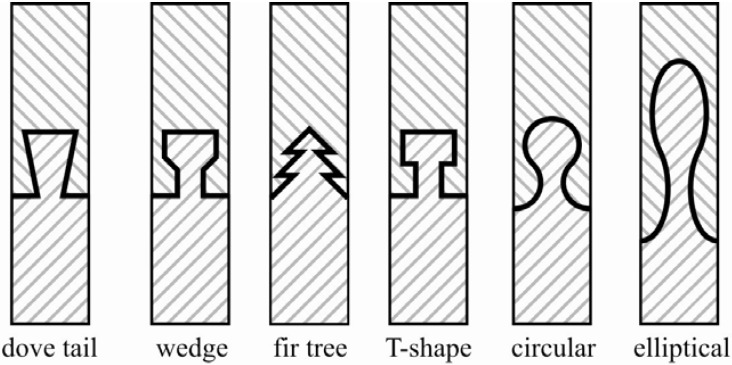
Elementary shapes of basic connecting elements.

In technical solutions—especially in woodwork and plastics engineering—a lot of different undercutting elements are known. So a lot of experience is available for traditional isotropic or medium anisotropic materials. Based on these, elementary shapes for connecting elements were deduced (Figure 2).

In real composite components, these connecting elements mainly have to transfer in-plane-forces. So they have to be designed for fiber-parallel loads and in-plane shear loads. The undercutting geometry has to be adapted to the anisotropic material behavior. The load-transferring fibers have to remain uncut as much as possible. Furthermore, the areas of shear load transfer have to be large due to the low shear strength parallel to the fibers. In addition, the contour of the undercutting connection needs to be tangential to a big curvature radius to avoid notch stresses around the edges.

To find an optimized shape for the interlock basic connecting elements, the selection was performed in three steps. Out of a pool of six basic geometries, the best three were selected considering aspects of the size of the load transferring area from undercut to root, fiber adapting potential, contact pressure, notching effect, manufacturing, and bonding area. The six criteria were weighted against each other according to established design methods. The main focus was on criteria with respect to a fiber adapted design. Manufacturing and bonding issues were of minor importance. The criteria assessment is shown in Table 1. Three points are given if the criterion I is more important than II and one point for the opposite. Two points are given for the same weight. The value *W* describes the importance of the different criteria.

**Table 1:** Weighting of the different criteria.

			II						S	W
			A	B	C	D	E	F		
**I**	Undercut root area	A		2	3	3	3	3	14	**0.78**
	Fiber adapting potential	B	2		2	2	3	3	12	**0.67**
	Contact pressure	C	1	2		2	3	3	11	**0.61**
	Notching effect	D	1	2	2		3	3	11	**0.61**
	Manufacturing	E	1	1	1	1		2	6	**0.33**
	Bonding area	F	1	1	1	1	2		6	**0.33**

For each of the first four elementary designs in Figure 2, a second design for undercut connecting elements with slight modifications like notch radius or width were evaluated. Finally, the ten variants were rated according to the weighted design criteria (Table 2). Each of the ten variants is valued for every criterion with 0 points up to 4 points. 0 points stands for the lowest and 4 for the highest degree of fulfillment. So it can be seen, that the elliptical and circular shape of the interlock-connecting elements and the dove tail with smooth notches fulfill the criteria best and were further investigated in detail by means of finite element analysis (FEA) and experimental studies.

**Table 2:** Evaluation of the elementary designs for undercutting elements.

		Dove tail				Wedge				Fir tree				T-shape				Interlock			
		no mod.		smooth notches		no mod.		smooth notches		thin		thick		thick		thin		circle		elliptic	
	W	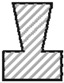		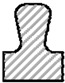		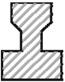		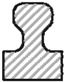		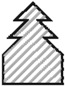		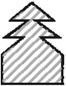		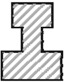		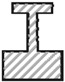		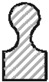		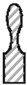	
		P	P·W	P	P·W	P	P·W	P	P·W	P	P·W	P	P·W	P	P·W	P	P·W	P	P·W	P	P·W
A	0.78	4	3.1	3	2.3	3	2.3	2	1.6	3	2.3	3	2.3	3	2.3	1	0.8	3	2.3	3	2.3
B	0.67	4	2.7	4	2.7	3	2.0	3	2.0	2	1.3	1	0.7	2	1.3	1	0.7	3	2.0	4	2.7
C	0.61	3	1.8	2	1.2	3	1.8	3	1.8	4	2.4	4	2.4	4	2.4	4	2.4	3	1.8	2	1.2
D	0.61	1	0.6	3	1.8	1	0.6	3	1.8	0	0.0	0	0.0	1	0.6	1	0.6	3	1.8	4	2.4
E	0.33	2	0.7	3	1.0	2	0.7	3	1.0	0	0.0	0	0.0	1	0.3	1	0.3	4	1.3	4	1.3
F	0.33	2	0.7	2	0.7	3	1.0	3	1.0	4	1.3	4	1.3	3	1.0	3	1.0	2	0.7	3	1.0
			**9.6**		**9.7**		**8.4**		**9.2**		**7.4**		**6.8**		**8.1**		**5.8**		**10.0**		**11.0**

The simulation was done within the FEA software Abaqus (V8.6-1) under utilization of a material sub-routine (Umat). For a comparative assessment of the selected basic undercutting design elements, the results of the two dimensional models were interpreted by using the Cuntze failure criterion.

**Figure 3:**
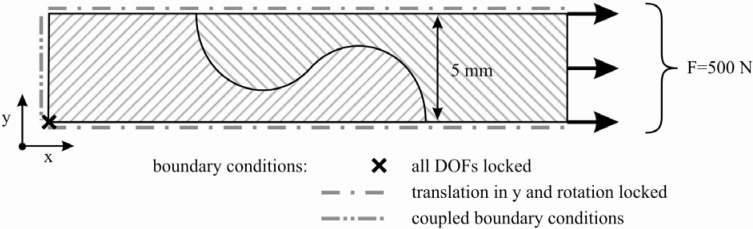
Boundary conditions used for the two dimensional simulation.

According to performed tension tests, the load was induced parallel to the fibers in 0°-direction. Because of the repetitive and symmetric shape of the elements, the modeling of only one half of the basic undercutting element—representing one fifth of the width of the tensile specimen—was possible. Figure 3 shows the simulated section with its coupled boundary conditions and the applied load. These boundary conditions are also feasible for the specimen edges due to a closed contour under realistic repair conditions.

**Figure 4:**
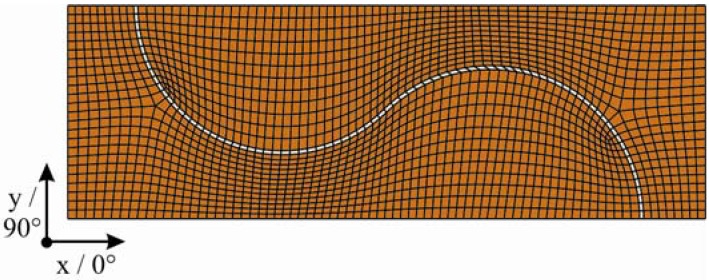
FE-mesh used for the two-dimensional preliminary design.

For the preliminary design, an efficient model with a mesh of four-node shell elements was designed (Figure 4). The thickness is 1.2 mm, according to the [0/90]_S_ laminate architecture. Table 3 shows the material properties used as input parameters. The modeling of the bonding layer—which is necessary for the adhesive connection of the interlock partners—was carried out by using one row of elements with isotropic material properties shown in Table 4. This simplified modeling of the bond line is feasible due to their minor importance for the load bearing behavior of the interlock connection, especially in case of the present basic investigation of different interlock geometries.

**Table 3:** Material properties of the prepreg laminate system [20].

Property		Value
Fibers		AS4
Matrix		3501-6 ep.
Fiber volume fraction	ϕ_f_	0.60
Longitudinal modulus	E_||_	126 GPa
Transverse modulus	E_⊥_	11 GPa
In-plane shear modulus	G_||⊥_	6.6 GPa
Major Poisson’s ratio	ν	0.28
Through-thickness Poisson’s ratio	ν_⊥⊥_	0.4
Longitudinal tensile strength	R^t^_||_	1950 MPa
Longitudinal compressive strength	R^c^_||_	1480 MPa
Transverse tensile strength	R^t^_⊥_	48 MPa
Transverse compressive strength	R^c^_⊥_	200 MPa
In-plane shear strength	R_⊥||_	79 MPa

**Table 4:** Material properties used for the bonding line.

Property		Value
Matrix type		MY750
Young’s modulus	E_m_	3.35 GPa
Shear modulus	G_m_	1.24 GPa
Major Poisson’s ratio	ν_m_	0.35
Tensile strength	R^t^_m_	80 MPa
Compressive strength	R^c^_m_	120 MPa

**Figure 5:**
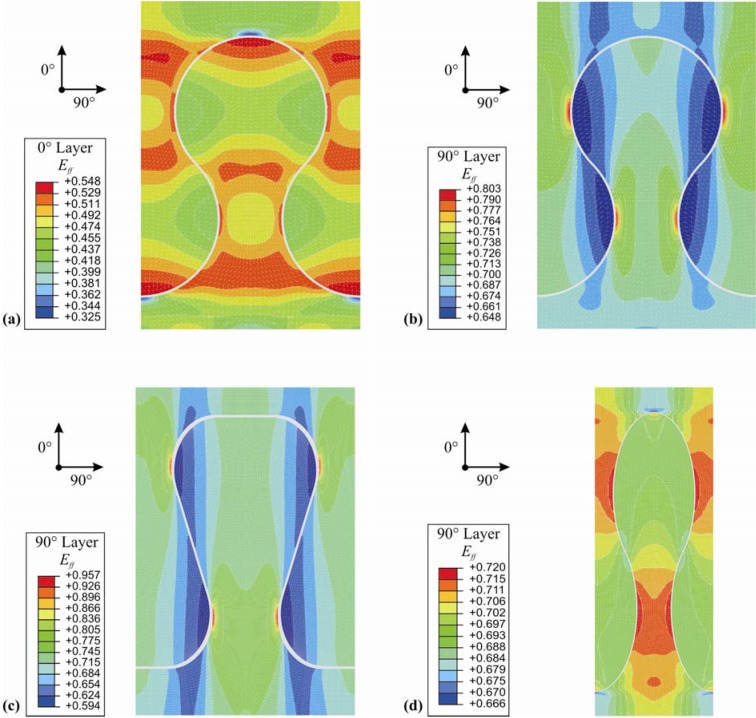
Resultant stress effort for (**a**) circular interlock in 0°-layer; (**b**) circular interlock in 90°-layer; (**c**) dove tail interlock in 90°-layer; (**d**) elliptical interlock in 90°-layer.

The analysis of the simulation results was done using Cuntze’s mode specific efforts in its individual fracture types. The resultant stress effort in each laminate ply can be determined from the individual stress efforts. Figure 5 shows the representative resultant stress effort *E_ff_* for fiber directions of the two circular interlocks with a circle radius of 3.5 mm as well as the comparable results in 90°-direction for dove tail and elliptical interlock connections. It can be seen that *E_ff_* in the 90°-layer is 45% higher than in the 0°-layer. As expected, the most critical area is the small counter section (neck). For all analyzed geometries it was assessed that the inter-fiber failure (matrix failure due to normal stress) in the 90°-layer is the most critical one.

In order to investigate the influence of the shape-specific geometrical parameters, extensive parameter studies were accomplished. Especially due to the minimum stress effort of circular and elliptical interlock connections, a parameter study regarding their optimum radii was performed. Table 5 shows the strong influence of the radius size on the resultant stress effort. For the width of 10 mm for one interlock element, it was proven that a circular element reveals an optimum at a radius of 3.5 mm. The same value is chosen for the semi-minor axis of the elliptical variant, where the semi-major axis has to be 10 mm.

**Table 5:** Resultant stress efforts depending on the radius parameter of 10 mm wide interlock variants.

Geometry	*Parameter [mm]*	E_ff_ in 0°-direction	E_ff_ in 90°-direction	Sketch
Circular interlock	*2.0*	0.688	0.957	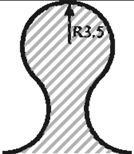
	*2.5*	0.602	0.912	
	*3.0*	0.581	0.846	
	*3.5*	**0.548**	**0.803**	
	*4.0*	0.556	0.824	
	*4.8*	0.695	0.966	
Elliptical interlock	*3.5 / 8.5*	0.598	0.789	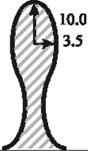
	*3.5 / 9.0*	0.588	0.766	
	*3.5 / 9.5*	0.575	0.758	
	*3.5 / 10.0*	**0.558**	**0.719**	
	*3.5 / 10.5*	0.565	0.749	
	*3.5 / 11.0*	0.572	0.762	

The variation of the undercutting angle of the dove tail did not show a significant effect. Finally, the circular as well as the elliptical interlock connection geometry exhibit the best performance for an undercutting connection element. Table 6 compares the resultant material efforts of the three analyzed geometries.

**Table 6:** Resultant stress efforts of the analyzed undercut shapes.

Geometry	*Parameters*		E_ff_ in 0°-direction	E_ff_ in 90°-direction	Sketch
Dove tail (smooth notches)	*Opening angle α radius r*	*35° 2.25 mm*	0.672	0.957	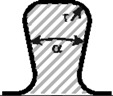
Circular interlock	*Radius*	*3.5 mm*	0.548	0.803	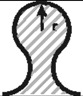
Elliptic interlock	*Major axis r_a_ Minor axis r_b_*	*10.0 mm 3.5 mm*	0.558	0.719	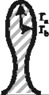

## 4. Experimental Work

**Figure 6:**
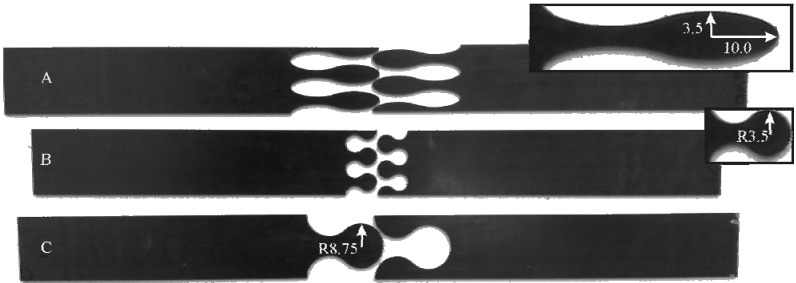
Tested interlock geometries.

For the experimental analysis of the developed composite repair technique based on undercutting elements by means of tensile tests, appropriate interlock specimens based on the simulation results were manufactured. As composite material for specimen preparation, a symmetric even laminate was chosen. It is composed out of four layers of unidirectional prepreg material with carbon fibers in0°- and 90°-direction and an epoxy matrix system. An elliptical and a circular geometry with two and a half interlock connection elements along the specimen width were selected. Additionally, a third geometry with an up-scaled circular element was analyzed (Figure 6). The specimens were manufactured by waterjet-cutting out of composite plates with a [0/90]_S_-lay-up. After cutting, the specimen halves were adhesively bonded by use of epoxy resin in order to ensure the exact edge alignment of the halves and consequently to avoid undefined multiaxial stresses.

**Figure 7:**
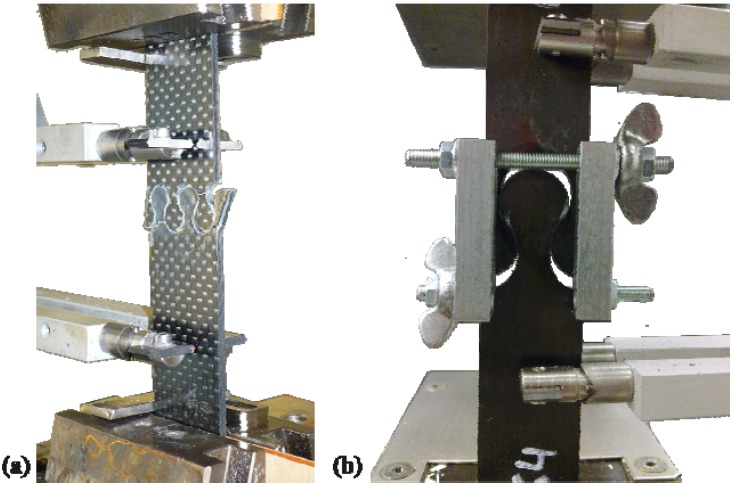
(**a**) Lateral bending of interlock elements; (**b**) Avoided lateral bending by using guiding element.

In addition to the three geometries, the influence of the lateral support was considered. In reality the damage of a structure will be mostly enclosed and surrounded by composite material. So the repair zone will not have free edges and the interlock base elements will always have a neighbor to avoid lateral bending, which occurs during the tensile tests (Figure 7). To simulate this support, subsequent tensile tests were performed by using an additional guiding element. It was mounted beside the tension rod so that it avoids lateral bending of the interlock elements but not the axial movement of the specimen.

**Figure 8:**
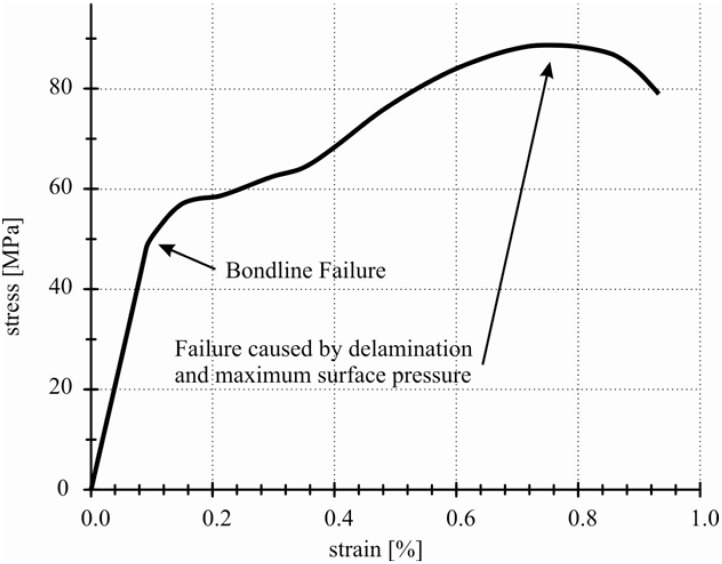
Typical failure shown in stress-strain diagram.

**Figure 9:**
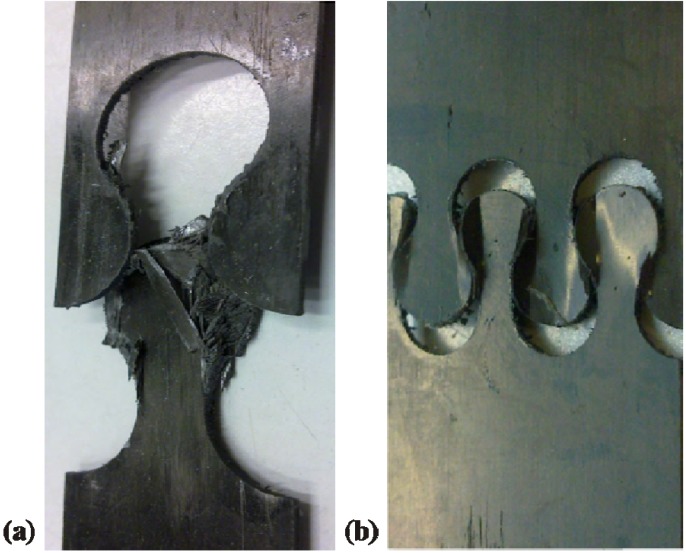
Typical failure of interlocks during tensile testing.

Basic failure phenomena appeared in every tested sample. As first phenomenon bond line cracking occurs, shown in form of curve flattening after the linear slope in the stress-strain diagram in Figure 8. After further increase of the tensile force, the laminate completely failed. This is caused by exceeding the geometry-specific maximum surface pressure of the connection interface. Additionally, a pull-through effect occurs. The single layers of one connection partner delaminate and split the single layers of the second partner (Figure 9).

**Figure 10:**
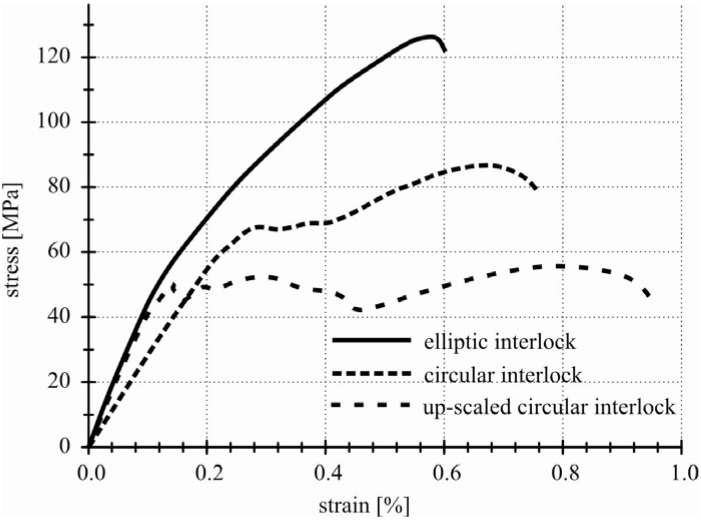
Stress-strain diagram of different interlock geometries.

The accomplished tests exhibit a significant geometry dependency of the stress-strain behavior. Compared to each other, the elliptical interlock geometry reached the highest values in stiffness and tensile strength (Figure 10).

## 5. Conclusions

The introduced novel composite repair technology based on undercutting interlock elements exhibits excellent possibilities with regard to a fully automated repair process, especially for large outer skin panels with visible surfaces and one-sided accessibility.

Fundamental numerical analyses make a contribution to the subsequent geometry definition and parameter adaptation of the interlock connection elements. Based on the simulation results, extensive experimental repair studies and tensile tests were accomplished. The findings show the best load bearing behavior for interlock elements with elliptical shape. In consequence of the geometry modification compared to circular interlock elements of different sizes, a doubling of the tensile strength was achieved. In addition, the interlock repair design indicates a non-problematic quasi-ductile failure behavior due to the specific subsequent delamination progress.

The repaired tensile specimen showed a structural stiffness of about 30 % compared to the untreated laminate. In order to analyze the highly geometry-dependent mechanical properties of the interlock repair zone, further studies on thick-walled large-scale composite structures are planned. Due to the global structural support of the interlock zone and the thick laminate, much higher mechanical stiffness and strength compared to the analyzed tensile specimen are expected.
